# Co-expression of Dorsal and Rel2 Negatively Regulates Antimicrobial Peptide Expression in the Tobacco Hornworm *Manduca sexta*

**DOI:** 10.1038/srep20654

**Published:** 2016-02-05

**Authors:** Xue Zhong, Xiang-Jun Rao, Hui-Yu Yi, Xin-Yu Lin, Xiao-Hong Huang, Xiao-Qiang Yu

**Affiliations:** 1 Division of Molecular Biology and Biochemistry, School of Biological Sciences, University of Missouri-Kansas City, 5007 Rockhill Road, Kansas City, MO 64110, USA; 2 Department of Entomology, School of Plant Protection, Anhui Agricultural University, Hefei, Anhui 230036, China; 3 College of Animal Science, Fujian Agriculture and Forestry University, Fuzhou, Fujian 350002, China

## Abstract

Nuclear factor κB (NF-κB) plays an essential role in regulation of innate immunity. In mammals, NF-κB factors can form homodimers and heterodimers to activate gene expression. In insects, three NF-κB factors, Dorsal, Dif and Relish, have been identified to activate antimicrobial peptide (AMP) gene expression. However, it is not clear whether Dorsal (or Dif) and Relish can form heterodimers. Here we report the identification and functional analysis of a Dorsal homologue (MsDorsal) and two Relish short isoforms (MsRel2A and MsRel2B) from the tobacco hornworm, *Manduca sexta*. Both MsRel2A and MsRel2B contain only a Rel homology domain (RHD) and lack the ankyrin-repeat inhibitory domain. Overexpression of the RHD domains of MsDorsal and MsRel2 in *Drosophila melanogaster* S2 and *Spodoptera frugiperda* Sf9 cells can activate AMP gene promoters from *M. sexta* and *D. melanogaster*. We for the first time confirmed the interaction between MsDorsal-RHD and MsRel2-RHD, and suggesting that Dorsal and Rel2 may form heterodimers. More importantly, co-expression of MsDorsal-RHD with MsRel2-RHD suppressed activation of several *M. sexta* AMP gene promoters. Our results suggest that the short MsRel2 isoforms may form heterodimers with MsDorsal as a novel mechanism to prevent over-activation of antimicrobial peptides.

Nuclear factor κB (NF-κB) family of transcription factors plays essential roles in regulating expression of immune-related genes^1^,^2^. These factors contain an N-terminal Rel homology domain (RHD) that can interact with DNA. There are two classes of NF-κB factors in mammals. Class I NF-κB factors include p105 and p100, which contain an N-terminal RHD and a C-terminal long inhibitory ankyrin repeats that must be cleaved off to activate gene expression. Class II NF-κB factors include RelA (p65), RelB and c-Rel that contain an N-terminal RHD and a C-terminal transactivation domain^3^. NF-κB factors can form homodimers and heterodimers in the nucleus, which bind to NF-κB DNA elements in the promoter regions of many immune-related genes^2^. In *Drosophila melanogaster*, three NF-κB factors, Dorsal, Dorsal-related immunity factor (Dif) and Relish, have been identified^4^. Dorsal and Dif belong to the Class II NF-κB factors and they are involved in the Toll pathway to regulate dorsal-ventral patterning during embryonic development^5^ or expression of antimicrobial peptides (AMPs) such as drosomycin in *Drosophila* larvae and adults^6^,^7^,^8^. Relish is a member of the Class I NF-κB factors and is cleaved to release the N-terminal fragment containing RHD upon activation of the immune deficiency (IMD) pathway^9^,^10^. Relish also regulates expression of AMPs including diptericin^11^. Synthesis of AMPs is one of the major defense mechanisms in insects^12^,^13^,^14^. The Toll pathway mediates immune responses against most Gram-positive bacteria and fungi^15^, while the IMD pathway is activated by Gram-negative bacteria^16^. It has been suggested that *Drosophila* Dif and Relish may form heterodimers to synergistically increase AMP production^17^,^18^.

NF-κB factors have been identified in the phylum of arthropoda^19^,^20^,^21^,^22^,^23^,^24^,^25^,^26^,^27^,^28^,^29^,^30^,^31^. In the mosquito *Aedes aegypti*, two Dorsal isoforms, AaREL1-A and AaREL1-B, can cooperatively enhance activation of immune genes^22^, and three alternatively spliced isoforms of Relish are generated from a single inducible Relish gene^21^. The *Anopheles gambiae* REL2 gene produces two spliced forms: a full-length REL2-F and a shorter REL2-S^32^. In the silkworm *Bombyx mori*, two Rel (Dorsal) proteins, BmRelA and BmRelB, activate antimicrobial peptide genes differently^24^, and two Relish homologs (BmRelish1 and 2) have also been identified^23^, and BmRelish2 isoform is a dominant negative regulator of the active BmRelish1^23^. In the pacific white shrimp *Litopenaeus vannamei*, LvRelish and its short isoform (sLvRelish) have been identified^19^.

The tobacco hornworm, *Manduca sexta*, is a popular lepidopteran model organism for study of insect immunity^33^. Various immune-related genes have been identified and characterized^34^, including AMP genes moricin, lebocin and gloverin^35^,^36^,^37^; C-type lectins^38^,^39^,^40^,^41^,^42^; and β-1,3-glucan recognition proteins^43^,^44^,^45^,^46^. A Toll-Spätzle pathway has been confirmed in *M. sexta* and it plays an important role in immune responses^47^. Previously, we reported that *M*. *sexta* moricin promoter contains both NF-κB and GATA elements^48^. Although the roles of NF-κB factors in regulation of gene expression in *M*. *sexta* have been proposed^49^, there have been no functional studies of NF-κB factors so far. Here we report cloning and functional studies of three *M. sexta* NF-κB homologs. The two short isoforms of *M. sexta* Relish, named MsRel2A and MsRel2B, contain only an RHD domain and lack the ankyrin-repeat inhibitory domain. Both MsRel2A and MsRel2B can activate AMP gene promoters. More importantly, we confirmed interaction of MsDorsal with MsRel2 for the first time, and suggesting that MsDorsal may form heterodimers with MsRel2. We also showed for the first time that co-expression of MsDorsal and MsRel2 suppressed the expression of AMP gene promoters. Our results suggest that active Relish short isoforms such as MsRel2A and MsRel2B can activate AMP genes as homodimers, and they may also form heterodimers with MsDorsal as a novel mechanism to negatively regulate AMP gene expression to prevent over-activation of AMPs.

## Results

### Cloning and sequence analysis of *M. sexta* Dorsal and Rel2

Based on the partial sequences from the *M. sexta* EST database, we performed PCR amplification and RACE to obtain the full-length cDNAs of two *M. sexta* Relish isoforms, MsRel2A (GenBank accession number: HM363513) and MsRel2B (GenBank accession number: HM363514), and a Dorsal homologue (GenBank accession number: HM363515). MsRel2A cDNA is 1677 bp long with an opening reading frame (ORF) of 1191 bp, which encodes a putative protein of 397 amino acids. MsRel2B cDNA is 2057 bp with an ORF of 1326 bp encoding a putative protein of 442 amino acids. MsRel2A and MsRel2B have an identical Rel homology domain (RHD) and only differ at the C-terminal regions. MsRel2A and MsRel2B share 91.7% identity, but MsRel2B is 45 amino acids longer at the C-terminus. MsDorsal RHD is 263 amino acids long. Sequence analysis showed that MsDorsal-RHD is most similar to RHDs of the class II NF-κB, while MsRel2-RHD is most similar to RHDs of class I NF-κB (Fig. S1A and S1B). Both MsRel2A and MsRel2B lack the ankyrin-repeat inhibitory domain, which is presence in the full-length Relish.

### Expression profile of *M. sexta* Dorsal and Rel2

**Figure 1:**
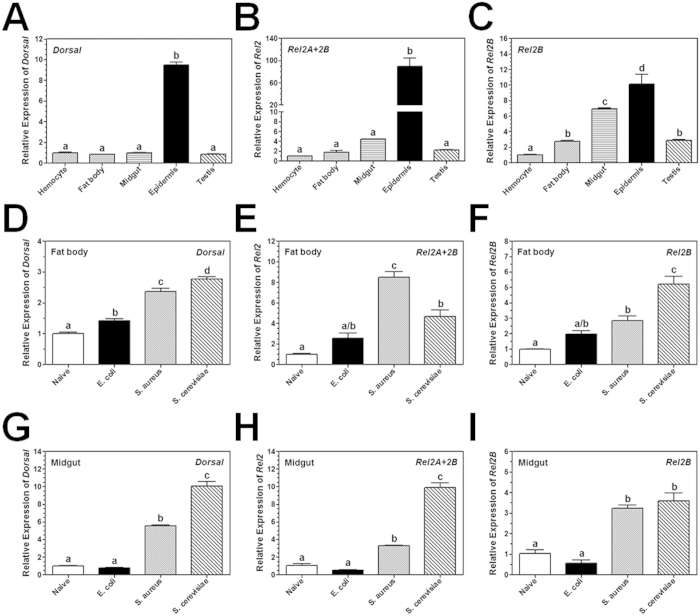
Tissue profile and induced expression of *M. sexta Dorsal* and *Rel2*. Total RNA samples were prepared from different tissues of naïve larvae (**A–C**), or from fat body and midgut of naïve larvae or larvae injected with *E. coli*, *S. aureus* and *S. cerevisiae* (**D–I**), and cDNA was prepared by reverse transcription. Real-time PCR was performed for *MsDorsal*, *MsRel2*, and *MsRel2B* using ribosomal protein S3 (*rpS3*) gene as an internal standard.

**Figure 2:**
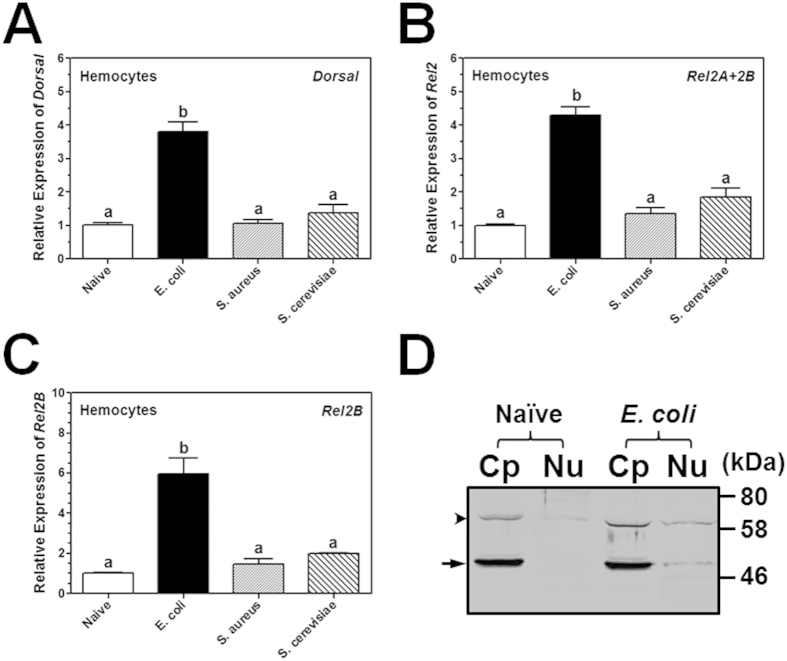
Induced expression of *M. sexta* Dorsal and Rel2 in hemocytes. Total RNA samples were prepared from hemocytes of naïve larvae or larvae injected with *E. coli*, *S. aureus* and *S. cerevisiae* (**A–C**), and real-time PCR was performed for *MsDorsal*, *MsRel2* and *MsRel2B* as described in Fig. 1. Cytoplasmic (Cp) and nuclear (Nu) proteins were also extracted from hemocytes of naïve and *E. coli*-injected larvae, and proteins were separated on 12% SDS-PAGE. MsDorsal in the protein extracts was detected by Western blot analysis using rabbit polyclonal antibody to recombinant MsDorsal-RHD (**D**). Arrow indicates MsDorsal, while arrow head indicates a possible MsDorsal dimer or non-specific protein band recognized by anti-MsDorsal antibody.

Tissue distribution profile of *MsDorsal*, *MsRel2A* and *MsRel2B* in *M. sexta* naïve larvae was determined by real-time PCR. Since *MsRel2A* and *MsRel2B* cDNA sequences are highly identical, we cannot design primers specific for *MsRel2A*. But *MsRel2B* cDNA is longer than *MsRel2A* at the 3′ end. Thus, we designed primers for *MsRel2* (*MsRel2A* + *MsRel2B*) and *specific primers for MsRel2B in real-time* PCR reactions. The results showed that *MsDorsal* and *MsRel2* mRNAs were highly expressed in epidermis compared to other tissues (hemocytes, fat body, midgut and testis), and only *MsRel2B* was also expressed at a high level in the midgut (Fig. 1A–C). To determine induced expression of these NF-κB factors by microbial infection, *M. sexta* larvae were injected with *Staphylococcus aureus*, *Escherichia coli* and *Saccharomyces cerevisiae*, and *MsDorsal* and *MsRel2* transcripts were measured by real-time PCR. Compared to the naïve larvae, expression of *MsDorsal*, *MsRel2* and *MsRel2B* mRNAs in fat body, midgut and hemocytes was significantly induced by injection of microorganisms, but the overall induced expression level was not high, and the induction of individual NF-κB factor genes depends on the tissues and microorganisms injected (Figs 1D–I and 2A–C). To test whether MsDorsal protein is also induced by microbial injection, Western blot was performed for cytoplasmic and nuclear protein extracts from hemocytes of naïve and *E. coli*-injected *M. sexta* larvae. The results showed that MsDorsal protein was detected at high levels in the cytoplasmic proteins of both naïve and *E. coli*-injected larvae (Fig. 2D, lanes Cp, arrow head); however, MsDorsal protein was detected only in the nuclear proteins of *E. coli*-injected larvae but not in the nuclear proteins of naïve larvae (Fig. 2D, lanes Nu), suggesting that bacterial infection can induce translocation of MsDorsal from the cytoplasm to the nucleus to activate gene expression.

### MsRel2 and MsDorsal activate promoter activity of *M. sexta* AMP genes

**Figure 3:**
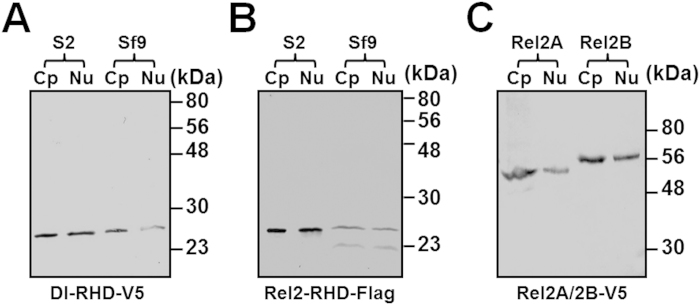
Western blot analysis of recombinant MsDorsal-RHD, MsRel2-RHD, MsRel2A and MsRel2B in S2 and Sf9 cells. V5-tagged MsDorsal-RHD, Flag-tagged MsRel2-RHD, V5-tagged MsRel2A and MsRel2B (full-length proteins) were transiently expressed in S2 or Sf9 cells, and cytoplasmic (Cp) and nuclear (Nu) proteins were prepared separately for Western blot analysis using monoclonal anti-V5 or anti-Flag antibody. Panels A and B: DI-RHD-V5 and Rel2-RHD-Flag, respectively; Panel C: MsRel2A-V5 and MsRel2B-V5 from S2 cells.

**Figure 4:**
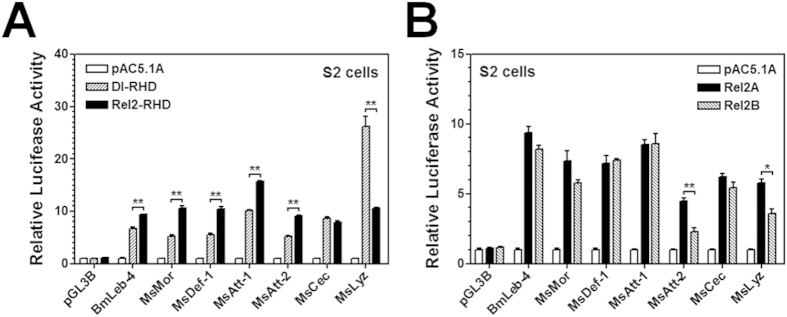
MsDorsal and MsRel2 can activate *M. sext*a AMP gene promoters. Relative luciferase activity of *M. sexta* AMP gene promoters activated by MsDorsal-RHD and MsRel2-RHD (**A**), or by MsRel2A and MsRel2B (**B**) in S2 cells was measured by Dual-Luciferase^®^ Reporter Assay System as described in the Materials and Methods. Bars represent the mean of three independent measurements ±SEM.

*D. melanogaster* and *M. sexta* AMP genes can be significantly upregulated by various microbial components^50^. Since NF-κB transcription factors play central roles in activation of AMP genes, we carried out dual-luciferase reporter assays in both *D. melanogaster* S2 cells and *Spodoptera frugiperda* Sf9 cells to determine whether MsDorsal and MsRel2 can activate different AMP gene promoters, as *M. sexta* and *D. melanogaster* AMP gene promoters are activated differently in S2 and Sf9 cells^48^. Recombinant MsDorsal-RHD (Dl-RHD) and MsRel2-RHD (Rel2-RHD) (only the RHD domains), as well as MsRel2A and MsRel2B (full length proteins) were successfully expressed in both S2 cells and Sf9 cells (Fig. 3), which can be detected in both the cytoplasm (Fig. 3, lanes Cp) and the nucleus (Fig. 3, lanes Nu). Overexpression of recombinant Dl-RHD and Rel2-RHD in S2 cells can significantly activate promoter activity of several *M. sexta* AMP gene promoters and *B. mori* lebocin-4 promoter (Fig. 4A). Most AMP gene promoters, including *M. sexta* moricin, defensin-1 and two attacins, and *B. mori* lebocin-4, were activated to significantly higher levels by Rel2-RHD than by Dl-RHD, while *M. sexta* cecropin promoter was activated to an equally high level by Dl-RHD and Rel2-RHD, but *M. sexta* lysozyme promoter was activated to a significantly higher level by Rel2-RHD than by Dl-RHD (Fig. 4A). Similarly, overexpression of the full length Rel2A and Rel2B can also significantly stimulate the activity of AMP gene promoters, and Rel2A and Rel2B had similar activity in activation of most AMP gene promoters (except *M. sexta* attacin-2 and lysozyme) (Fig. 4B).

### Activation of moricin and lysozyme promoters by MsDorsal and MsRel2

**Figure 5:**
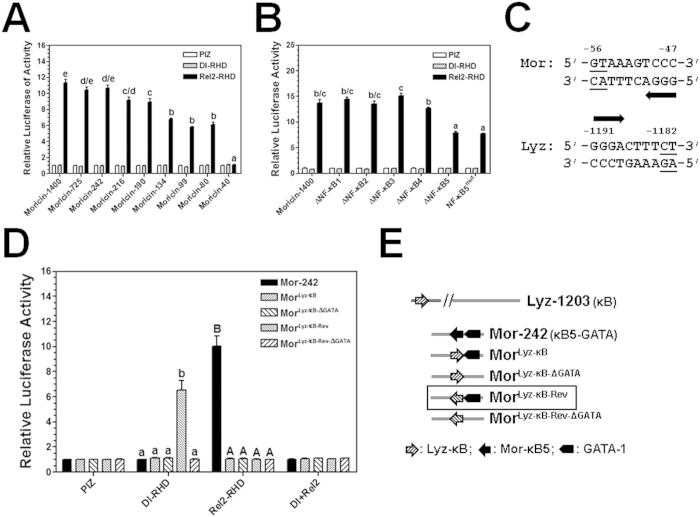
Moricin promoter is activated mainly by MsRel2-RHD. The relative luciferase activities of different truncated moricin promoters (**A**) or different NF-κB-deleted or mutated moricin promoters (**B**) activated by recombinant MsDorsal-RHD (Dl-RHD) or MsRel2-RHD (Rel2-RHD) in Sf9 cells, or different constructs of moricin promoters (see panel **E**) activated by recombinant MsDorsal-RHD, MsRel2-RHD, or the two RHDs together (Dl + Rel2) in Sf9 cells (**D**) were determined by Dual-Luciferase^®^ Reporter Assay System as described in the Materials and Methods. (**C**) The nucleotide sequences of the NF-κB sites from *M. sexta* moricin (Mor) and lysozyme (Lyz) promoters. The arrows indicate the direction of NF-κB sites. (**E**) Schematic diagrams of the moricin promoters (see text for detail information). Bars represent the mean of three independent measurements ±SEM.

We have previously characterized a moricin promoter (1400 bp) and also cloned a lysozyme promoter (1203 bp) in *M. sexta*. Five NF-κB sites were predicted in the MsMoricin promoter, but only the proximal NF-κB5 (Mor-NF-κB5) is activated in Sf9 cells by peptidoglycan from *E. coli*^48^. To test whether MsMoricin can be activated by MsDorsal and/or MsRel2 and whether the five predicted NF-κB sites are all functionally active, reporter luciferase assays were performed with MsMoricin promoter and its deletion and mutation promoters in Sf9 cells since MsMoricin promoter showed low activity in S2 cells but high activity in Sf9 cells^48^. All the MsMoricin promoters showed almost no activity in Sf9 cells after overexpression of MsDorsal-RHD (Fig. 5A,B), indicating that MsMoricin is not activated by Dorsal. MsMoricin (1400 bp), MsMoricin-725 (725 bp) and MsMoricin-242 (242 bp) promoters were activated to similar high levels by MsRel2-RHD, but MsMoricin-40 (40 bp) promoter did not have any activity (Fig. 5A). Deletion of the predicted NF-κB1, NF-κB2, NF-κB3 or NF-κB4 site did not have an effect on the activity of MsMoricin-242 promoter activated by MsRel2-RHD, but deletion or mutation of NF-κB5 site significantly decreased the activity of MsMoricin-242 promoter (Fig. 5B), indicating that MsMoricin is activated by Rel2 and only the NF-κB5 site is functionally active.

We showed that MsLysozyme promoter was activated by MsDorsal-RHD (Fig. 4A), and only one NF-κB site was predicted in the lysozyme promoter. Moricin NF-κB site differs from lysozyme NF-κB site only at two 3′ nucleotides but the two NF-κB sites have opposite direction (Fig. 5C). To test whether the consensus sequence and direction of NF-κB sites as well as other transcription factor binding sites are required for activation of AMP gene promoters by Dorsal and Rel2, we made several mutations in the Mor-242 promoter (242 bp) by replacing NF-κB5 site with lysozyme NF-κB site (Lyz-κB, with an opposite direction to NF-κB5) or reversed lysozyme NF-κB site (Lyz-κB-Rev, with the same direction to NF-κB5) and with or without GATA-1 site (Fig. 5E), since GATA-1 is required for NF-κB5 to activate moricin promoter^48^. It has also been reported that *Drosophila* Dif and Relish may form heterodimers to synergistically activate AMPs^17^. Thus, we also test whether co-expression of MsDorsal-RHD and MsRel2-RHD has an effect on the activity of the moricin promoters. Among the five moricin promoters, only Mor-242 promoter (containing both NF-κB5 and GATA-1 sites) was activated by MsRel2-RHD, and only Mor^Lyz-κB-Rev^ promoter (containing both the reversed lysozyme NF-κB site that has the same direction to NF-κB5 and GATA-1) was activated by MsDorsal-RHD, but none of the five promoters was activated by co-expression of MsRel2-RHD and MsDorsal-RHD (Fig. 5D).

**Figure 6:**
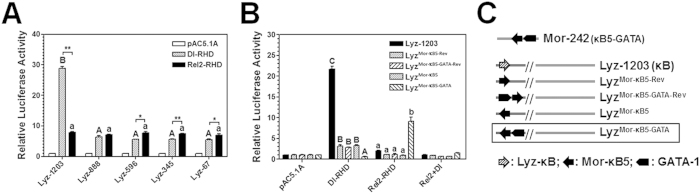
Lysozyme promoter is activated mainly by MsDorsal-RHD. The relative luciferase activities of different truncated lysozyme promoters (**A**) or different constructs of lysozyme promoters (**B**) activated by recombinant MsDorsal-RHD, MsRel2-RHD, or the two RHDs together (Dl + Rel2) in S2 cells were determined by Dual-Luciferase^®^ Reporter Assay System. (**C**) Schematic diagrams of the lysozyme promoters (see text for detail information). Bars represent the mean of three independent measurements ±SEM.

To determine activation of MsLysozyme promoter by Dorsal and Rel2, we constructed four deletion promoters and four mutation promoters by replacing lysozyme NF-κB with moricin NF-κB5 (opposite direction to lysozyme NF-κB) or reversed NF-κB5 (same direction to lysozyme NF-κB) with or without GATA-1 site (Fig. 6C). Since lysozyme promoter showed similar high activities in both *Drosophila* S2 and *S. frugiperda* Sf9 cells^48^, activation of lysozyme promoters was performed in S2 cells. The results showed that only the lysozyme promoter (1203 bp) but not the four deletion promoters was activated by MsDorsal-RHD, and all five lysozyme promoters showed low basal activities when MsRel2-RHD was overexpressed (Fig. 6A), indicating that the distal lysozyme NF-κB is functional active and it binds to Dorsal but not Rel2. Among the lysozyme promoter and the four mutated promoters, only lysozyme promoter was activated by MsDorsal-RHD, and only Lyz^Mor-κB5-GATA^ promoter (containing moricin NF-κB5 and GATA-1 sites) was activated by MsRel2-RHD, and none of the five lysozyme promoters was activated by co-expression of MsDorsal-RHD and MsRel2-RHD (Fig. 6B).

### Interaction of MsDorsal with MsRel2

**Figure 7:**
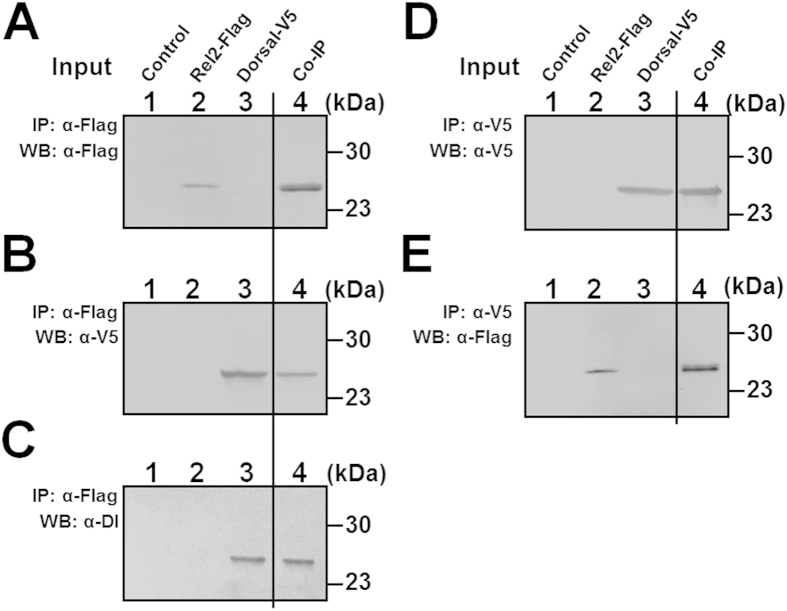
*M. sexta* Rel2-RHD interacts with Dorsal-RHD. Co-immunoprecipitation (Co-IP) assays were performed as described in the Materials and Methods. Immunoprecipitated (IP) proteins or Co-IP proteins were detected by immunoblotting using anti-Flag or anti-V5 monoclonal antibody (**A–E**), or anti-Dorsal polyclonal antibody (**C**) as the primary antibody. Lanes 1–3 from panels (**A–E**) were cell lysates alone (protein inputs) and lane 4 was IP and Co-IP proteins.

*Drosophila* Dif and Relish may form heterodimers and expression of peptide linked Dif-Relish-N (the N-terminal domain of Relish) can activate AMP genes in the Toll and IMD pathways^17^. We showed above that co-expression of MsDorsal-RHD and MsRel2-RHD abolished activation of moricin promoter by MsRel2-RHD (Fig. 5D) and lysozyme promoter by MsDorsal-RHD (Fig. 6B). To test interaction between MsDorsal and MsRel2, we over-expressed MsRel2-RHD-Flag and MsDorsal-RHD-V5 in S2 cells and performed co-immunoprecipitation (Co-IP) experiments. Expression of MsRel2-RHD-Flag and MsDorsal-RHD-V5 in S2 cells was confirmed by Western blot analysis with monoclonal anti-Flag or anti-V5 antibody (Fig. 7A,B,D,E, lanes 2 and 3 for input), and expression of MsDorsal-RHD-V5 was also confirmed by polyclonal rabbit anti-Dorsal antibody (Fig. 7C, lane 3). Since only the RHD domains of MsRel2 and MsDorsal were expressed and the two RHD domains have similar size but different tags, they were recognized by anti-V5 and anti-Flag antibodies, respectively, but appeared at the same location on the Western blot. Co-IP experiments showed that anti-Flag antibody can pull down MsRel2-RHD-flag (Fig. 7A, lane 4) and co-precipitated MsDorsal-RHD-V5, which was recognized by anti-V5 antibody (Fig. 7B, lane 4) and anti-Dorsal antibody (Fig. 7C, lane 4). Likewise, anti-V5 antibody can pull down MsDorsal-RHD-V5 (Fig. 7D, lane 4) and co-precipitated MsRel2-RHD-Flag, which was recognized by anti-Flag antibody (Fig. 7E, lane 4). These results suggest that *M. sexta* Dorsal and Rel2 may form heterodimers *in vivo*.

### Dorsal-Rel2 heterodimers as negative regulators in AMP gene expression

**Figure 8:**
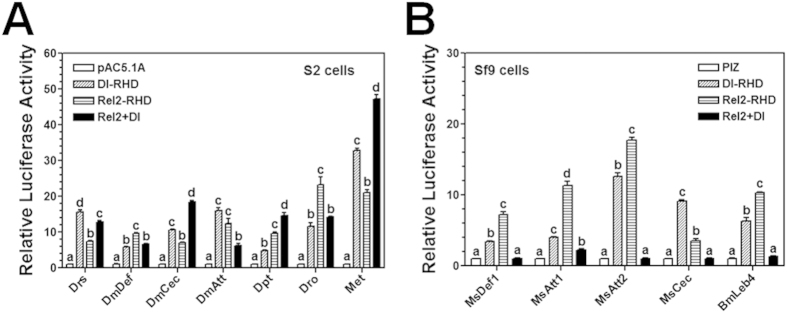
MsDorsal-RHD and MsRel2-RHD together negatively regulate *M. sexta* AMP gene promoters. MsDorsal-RHD (Dl-RHD) and MsRel2-RHD (Rel2-RHD) were expressed separately or together in S2 (**A**) or Sf9 (**B**) cells. Activation of *D. melanogaster* (**A**) or *M. sexta* (**B**) AMP gene promoters were determined by Dual-Luciferase^®^ Reporter Assay System. Drs, drosomycin; Def, defensin; Cec, cecropin; Att, attacin; Dpt, diptericin; Dro, drosocin; Met, metchnikowin; Leb, *B. mori* lebocin-4. Bars represent the mean of three independent measurements ±SEM.

We showed that co-expression of MsDorsal-RHD and MsRel2-RHD suppressed activation of moricin promoter by MsRel2-RHD (Fig. 5D) and lysozyme promoter by MsDorsal-RHD (Fig. 6B), respectively, and MsDorsal-RHD and MsRel2-RHD can interact with each other and may form heterodimers (Fig. 7). To test whether Dorsal-Rel2 heterodimers may also impact activation of other AMP gene promoters, luciferase assays were performed in S2 cells for *Drosophila* AMP gene promoters and in Sf9 cells for *M. sexta* AMP gene promoters after overexpression of MsDorsal-RHD or MsRel2-RHD alone, or co-expression of MsDorsal-RHD and MsRel2-RHD. Overexpression of MsDorsal-RHD or MsRel2-RHD alone can activate all the *Drosophila* and *M. sexta* AMP gene promoters to certain levels (Fig. 8). Co-expression of MsDorsal-RHD and MsRel2-RHD in S2 cells stimulated *Drosophila* cecropin, diptericin and metchnikowin promoters to significantly higher levels than by MsDorsal-RHD or MsRel2-RHD alone, but inhibited activation of attacin (Fig. 8A). However, co-expression of MsDorsal-RHD and MsRel2-RHD in Sf9 cells abolished activation of all the AMP gene promoters tested, including *M. sexta* defensin-1, attacin-1, attacin-2, cecropin, and *B. mori* lebocin-4 (Fig. 8B). These results suggest that in *M. sexta*, Dorsal-Rel2 heterodimers may serve as negative regulators in activation of AMPs.

## Discussion

In this study, we identified a Dorsal homolog (MsDorsal) and two short isoforms of Relish (MsRel2A and MsRel2B) in *M. sexta*, and investigated their roles in activation of AMP gene promoters. All three NF-κB factors were highly expressed in epidermis, but only *MsRel2B* was also expressed in the midgut. Expression of the three NF-κB factors in *M. sexta* larvae was induced in response to microbial infections depending on the tissues and microorganisms, but the induction levels were not high. Interestingly, *E. coli* injection induced translocation of MsDorsal from the cytoplasm to the nucleus, suggesting that induced translocation of NF-κB factors in the nucleus is the key to activate gene expression. MsDorsal, MsRel2A and MsRel2B were functionally active NF-κB factors that can activate *M. sexta* AMP gene promoters differently. Importantly, MsDorsal can interact with MsRel2 to form Dorsal-Rel2 heterodimers, which may serve as negative regulators in activation of *M. sexta* AMP genes, a novel mechanism to prevent over-activation of AMPs.

In mammals, NF-κB factors (p65, RelB, c-Rel, p50 and p52) can form homodimers and heterodimers to activate gene expression^51^. In *D. melanogaster*, it has been suggested that Dif and Relish may form heterodimers, and peptide linked Dif-Relish-N (N-terminal fragment of Relish) heterodimers can activate AMPs regulated by both the Toll and IMD pathways^17^. However, there has been no direct evidence for interaction of Dif (or Dorsal) with Relish, and peptide linked Dif-Relish-N dimers may not function as heterodimers. This is because the covalent linked Dif-Relish-N dimers may form non-covalent dimers of Dif-Relish-N dimers, in which Dif-Dif can be on one end, while Relish-N-Relish-N can be on the other end. Thus such non-covalent dimers of Dif-Relish-N heterodimers may still function as Dif-Dif and Relish-Relish homodimers. We for the first time demonstrated that MsDorsal-RHD and MsRel2-RHD can interact with each other and may form heterodimers, which suppressed the promoter activity of *M. sexta* AMP genes. By co-expressing MsDorsal-RHD and MsRel2-RHD in S2 and Sf9 cells, both homodimers of MsDorsal and MsRel2, as well as heterodimers of MsDorsal-MsRel2 can form. We already showed that homodimers of MsDorsal and MsRel2 can activate AMP gene promoters, therefore, suppression of the promoter activity of *M. sexta* AMP genes by co-expression of MsDorsal and MsRel2 in Sf9 cells must be due to formation of MsDorsal-MsRel2 heterodimers. Dorsal-Rel2 heterodimers as negative regulators in activation of AMP genes may be a new mechanism to prevent over-activation of the Toll and IMD pathways, since over-activation of AMPs and other immune-related genes can be detrimental to hosts^1^,^52^. We also noticed that co-expression of MsDorsal and MsRel2 in S2 cells only inhibited activation of *Drosophila* attacin promoter, but stimulated activation of cecropin, diptericin and metchnikowin promoters to significantly higher levels than by MsDorsal-RHD or MsRel2-RHD alone. This may be because activation of dipteran and lepidopteran AMP gene promoters in S2 cells (a dipteran cell line) and in Sf9 cells (a lepidopteran cell line) differs as demonstrated previously in our lab^40^, or *M. sexta* Dorsal and Rel2 proteins may not work the same way as *D. melanogaster* Dif (or Dorsal) and Relish. In addition, there has been no direct evidence for interaction of *Drosophila* Relish with Dif or Dorsal, and short isoforms of Relish have not been identified so far in *D. melanogaster*. Thus, it is not clear how Dif-Relish or Dorsal-Relish heterodimers are formed *in vivo* in *Drosophila*, since both the Toll and IMD pathways must be activated at the same time to release Dif (or Dorsal) and generate Relish-N for formation of heterodimers. Future work is to identify short isoforms of Relish, verify interaction between Relish-N and Dif (or Dorsal), and to determine regulation of AMP genes by NF-κB heterodimers in *Drosophila*.

In *D. melanogaster*, AMP genes are regulated by the Toll pathway via Dorsal/Dif and by the IMD pathway via Relish^53^,^54^,^55^. Based on the NF-κB sites of *Drosophila* AMP gene promoters, consensus sequences of NF-κB sites for Dorsal/Dif and Relish have been proposed^56^. In lepidopteran insects, components of the Toll and IMD pathways as well as NF-κB factors have been identified^57^,^58^, suggesting that the two signaling pathways in regulation of AMPs are conserved in lepidopteran insects. But it is not clear whether lepidopteran Dorsal and Relish proteins bind to the same NF-κB consensus sequences from *Drosophila*, and whether the direction of NF-κB sites as well as the non-consensus nucleotides also plays a role in selection/binding of Dorsal and Relish. Alignment of the active NF-κB sites from *Drosophila* and *M. sexta* AMP gene promoters showed that only the first three nucleotides (GGG) are highly conserved (Fig. S1C). We have identified two NF-κB sites with high selectivity for Relish (*M. sexta* moricin) and Dorsal (*M. sexta* lysozyme), respectively. Interestingly, the two NF-κB sites have opposite direction and only differ in two nucleotides at the 3′-end. By replacing the NF-κB site in the moricin promoter with the NF-κB site of lysozyme, and by replacing the NF-κB site in the lysozyme promoter with the NF-κB site of moricin in the presence or absence of GATA-1 site, we showed that both the direction and sequence (including the non-consensus sequence) of the NF-κB site are important for selection of NF-κB factors (Dorsal/Dif or Relish), and other transcription factors (for example, GATA-1 factor in moricin promoter) are also important for activation of AMP genes^48^,^56^,^59^. Thus, regulation of AMP gene expression by NF-κB factors in lepidopteran insects may differ from that in *Drosophila*. Future work is to compare gene regulation by NF-κB factors between dipteran and lepidopteran insects.

## Methods

### Insect rearing, D. melanogaster S2 and Spodoptera frugiperda Sf9 cell lines

*M. sexta* eggs were originally purchased from Carolina Biological Supplies (Burlington, NC, USA). Larvae were reared on an artificial diet at 25 °C^60^, and the fifth instar larvae were used for the experiments. *D. melanogaster* Schneider S2 cells were purchased from American Type Culture Collection (ATCC), and *Spodoptera frugiperda* Sf9 cells were purchased from Invitrogen (12552-014, Invitrogen).

### Cloning and sequence analysis of *M. sexta* Dorsal and Rel2 cDNAs

In the *M. sexta* EST library (http://entoplp.okstate.edu/profiles/jiang.htm), two EST fragments were predicted to encode Rel-homology domain (RHD)-containing proteins (manduca.Contig2427 and manduca.Contig7025). Gene specific primers were designed based on the EST sequences to clone the full-length cDNAs. Total RNA was prepared from the fat body of day 3 *M. sexta* naïve larvae using TRIzol^®^ Reagent (T9424, Sigma–Aldrich), and contaminated genomic DNA was removed by RQ1 RNase-free DNase I (Promega). Reverse transcription was performed using oligo(dT) primer (Promega) and ImProm-II reverse transcriptase (Promega) following the manufacturer’s instructions. The 5′ and 3′ RACE reactions were performed using smarter race kit (Clontech). The opening reading frame (ORF) was predicted from the nucleotide sequence using DNAMAN (Lynnon Corporation, Quebec, Canada). BLASTP (http://blast.ncbi.nlm.nih.gov/Blast.cgi) was used to search homologous RHD sequences. RHD sequences from various NF-κB factors were aligned with the MUSCLE module of MEGA 6.0. The aligned sequences were used to construct a neighbor-joining tree with 1000 Bootstrap Replications^61^. RHD sequences were aligned with Clustal Omega (http://www.ebi.ac.uk/Tools/msa/clustalo/), and the alignment result was decorated with ESPript 3.0^62^. Consensus sites of κB sites were displayed with WebLogo3^63^,^64^.

### Construction of luciferase reporter plasmids

To construct different mutated promoters, site-directed mutagenesis was performed using the wild-type *M. sexta* lysozyme promoter (1203-bp) and moricin deletion promoter (242-bp) as templates^48^. PCR program was 3 min at 95 °C, and then 17 cycles of 95 °C for 1 min, 55 °C for 2 min, 68 °C for 15 min, followed by a final extension at 68 °C for 30 min. The PCR products were recovered by agarose gel electrophoresis-Wizard^®^ SV Gel and PCR Clean-Up System (A9285, Promega), digested by *Dpn* I, and then transformed into competent *Escherichia coli* XL1 Blue cells. The mutant reporter plasmids were then purified and sequenced by an Applied Biosystems 3730 DNA Analyzer in the DNA Sequencing and Genotyping Facility at University of Missouri – Kansas City, and used for transient transfection in S2 or Sf9 cell lines.

### Tissue distribution and induced expression of *M. sexta* Rel2A, Rel2B and Dorsal

Hemocytes, fat body, midgut, epidermis and testis were collected from *M. sexta* day 2 fifth instar naïve larvae for preparation of cDNAs as described previously^47^ in 25 μl reactions using moloney murine leukemia virus (M-MLV) reverse transcriptase (M1701, Promega) with an anchor-oligo(dT)_18_ primer following the manufacturer’s instructions.

Hemocytes, fat body and midgut were also collected separately from day 2 fifth instar larvae injected with heat-killed *E. coli* strain XL1-blue (5 × 10^7 ^cells/larva), *Staphylococcus aureus* (5 × 10^7 ^cells/larva), or *Saccharomyces cerevisiae* (10^7 ^cells/larva) at 24 h post-injection for preparation of total RNA and cDNA. Real-time PCR was performed in 20 μl reactions containing 10 μl 2 × SYBR^®^ GreenER^™^ qPCR SuperMix Universal (No. 204141, Qiagen), 4 μl H_2_O, 4 μl diluted (1:50) cDNA, and 1 μl each reverse and forward diluted primer (10 pmol/μl), using the following program: 2 min at 50 °C, 10 min at 95 °C, followed by 40 cycles of 95 °C for 15 s, 60 °C for 1 min and the dissociation curve analysis. Data from three replicates of each sample was analyzed with SDS software (ABI) using a comparative method (2^−△△Ct^).

### Expression and purification of *M. sexta* Dorsal in bacteria and preparation of polyclonal rabbit antiserum

RT-PCR was performed to obtain cDNA sequence encoding MsDorsal-RHD domain (residues 92–263). The PCR fragment was ligated into the *Nco* I/*Xho* I digested expression vector pGEX-5X, and then transformed into competent *E. coli* BL21 (DE3) cells. Recombinant plasmids were prepared and confirmed by restriction enzyme digestion and DNA sequencing. To express and purify recombinant GST-MsDorsal-RHD fusion protein, overnight culture of a single bacterial colony in LB medium containing ampicillin (100 μg/ml) was diluted 1:100 in LB medium and incubated at 37 °C to OD_600_ = 0.8 and then isopropyl-D-thiogalactoside (IPTG) was added (at 0.5 mM final concentration) to induce protein expression. Recombinant protein was purified using Ni-NTA agarose beads (Qiagen) under native conditions following the manufacturer’s instructions. The purified recombinant GST-MsDorsal-RHD fusion protein was cleaved by thrombin, and the cleavage products were separated on 12% SDS-PAGE. The gel slice containing recombinant MsDorsal-RHD was used as an antigen to produce rabbit polyclonal antiserum at Cocalico Biologicals, Inc (Pennsylvania, USA).

### Construction of recombinant pAC5.1⁄V5-His A and pIZ/V5-His expression vectors

cDNA fragments encoding MsRel2A (residues 1–397), MsRel2B (residues 1–422), MsRel2-RHD (residues 58–227, identical for MsRel2A and MsRel2B), and MsDorsal-RHD (residues 92–263) were amplified by PCR. For pAC5.1⁄V5-His A vector (in S2 cells), forward primers for MsRel2A, MsRel2B and MsDorsal-RHD contain 5′ non-coding region recognized by *Drosophila* ribosome, followed by a start codon and a *Kpn* I site, while reverse primers contain an *Apa* I site. Forward primer for MsRel2-RHD contains a start codon and an *EcoR* I site and the reverse primer contains a *Not* I site followed by an in-frame Flag sequence and a stop codon. For pIZ/V5-His vector (in Sf9 cells), forward primers contain 5′ non-coding region suitable for Sf9 cell expression, followed by a start codon and a *Kpn* I site, while reverse primer for MsDorsal-RHD contains an *Xba* I site and the reverse primer for MsRel2-RHD contains an *Xba* I site followed by an in-frame Flag sequence and a stop codon. All primers are listed in Table S1. PCR reactions were performed with the following conditions: 94 °C for 3 min, 35 cycles of 94 °C for 30 s, Tm-5 °C for 30 s, 72 °C for 45 s to 90 s, followed by a final extension at 72 °C for 10 min. The PCR products were recovered and subcloned into *Kpn* I/*Apa* I, *EcoR*I/*Not*I, or *Kpn* I/*Xba* I digested pAC5.1⁄V5-His A or pIZ/V5-His vector (V413020, Invitrogen) using T4 DNA ligase (M0202L, NEB). Recombinant expression vectors were then purified and sequenced by an Applied Biosystems 3730 DNA Analyzer in the DNA Sequencing and Genotyping Facility at University of Missouri – Kansas City, and used for protein expression in S2 or Sf9 cell lines.

### Insect cell culture and Transient transfection

*D. melanogaster* Schneider S2 cells or *S. frugiperda* Sf9 cells were maintained at 27 °C in Insect Cell Culture Media (SH30610.02, Hyclone), supplemented with 10% heat-inactivated fetal bovine serum (#10082063, Invitrogen) containing 1% penicillin-streptomycin solution (G6784, Sigma-Aldrich). For DNA transfection, cells were placed overnight to 70% confluence prior to transfection in serum-free medium (SH30278.01, Hyclone). GenCarrier-1^TM^ transfection reagent (#31-00110, Epoch Biolabs) was used for transient transfection based on the manufacturer’s instructions. After 7 h transfection, S2 or Sf9 cells were centrifuged and resuspended in complete growth medium to induce protein expression for 48 h. The cell culture media and cell lysates were analyzed by Western blot.

### Western blot analysis and Co-immunoprecipitation (Co-IP) assay

For Western blot analysis of endogenous MsDorsal, hemocytes were collected from *M. sexta* fifth instar naïve larvae or larvae injected with heat-killed *E. coli* strain XL1-blue (5 × 10^7^ cells/larva) at 24 h post-injection. Nuclear and cytoplasmic proteins were extracted from hemocytes using nuclear extraction kit (Millipore, Cat. No. 2900). The concentrations of nuclear and cytoplasmic proteins were measured by Nano-Drop using BSA as the standard.

Western blot analysis was performed as described previously^47^ for cell culture media and cell lysates from S2 and Sf9 cells (2 × 10^6 ^cells/well). The cell culture media (10 μl each) and cell extracts (10 μl each, equivalent to ~5 × 10^4 ^cells) were separated on 10% or 12% SDS-PAGE and proteins were transferred to nitrocellulose membranes (162–0097, Bio-Rad). Anti-Flag M2 antibody (F-1804, Sigma-Aldrich, 1:5000 dilution) and anti-V5 antibody (V-8012, Sigma-Aldrich, 1:5000 dilution) were used as primary antibodies and alkaline phosphatase-conjugated anti-mouse antibody (A4312, Sigma-Aldrich, 1:10,000) was used as secondary antibody for color development. The signal was developed by alkaline phosphatase (AP)-conjugated color development Kit (#170–6432, Bio-Rad).

Co-immunoprecipitation (Co-IP) assays were performed the same as described previously using cell extracts containing recombinant proteins with anti-Flag M2 or anti-V5 antibody, and immunoprecipitated proteins were analyzed by immunoblotting^47^.

### Dual-Luciferase Reporter Assay

Dual-luciferase reporter assays in S2 and Sf9 cells were performed in 96-well culture plates with recombinant pAC5.1⁄V5-His A (in S2 cells) or pIZ/V5-His (in Sf9 cells) expression plasmid (0.3 μg), pGL3B (empty vector) or different pGL3B firefly luciferase reporter plasmids from the promoters of *M. sexta* or *D. melanogaster* antimicrobial peptide (AMP) genes, and several mutated *M. sexta* lysozyme or moricin promoters (0.15 μg), and renilla luciferase reporter plasmid (0.015 μg) (as an internal standard) (pRL-TK, Promega) the same as described previously^48^. Relative luciferase activity (RLA) from S2 or Sf9 cells co-transfected with empty pAC5.1⁄V5-His A or pIZ/V5-His and reporter vector was used as the calibrator.

### Statistical analysis of the data

At least three replicates of each sample were analyzed for each experiment, and experiments were repeated with three independent biological samples (or three independent cell cultures), and a typical set of data was used to make figures. Figures were made from means of three independent biological replicates with the GraphPad Prism. Significance of difference was determined by one way ANOVA followed by a Tukey’s multiple comparison tests using GraphPad Prism (GraphPad, CA). Identical letters are not significant difference (p > 0.05) while different letters indicate significant difference (p < 0.05) determined by one-way ANOVA followed by a Tukey’s multiple comparison test. Asterisks indicate significant difference (*p < 0.05; **p < 0.01) determined by one-way ANOVA.

## Additional Information

**How to cite this article**: Zhong, X. *et al.* Co-expression of Dorsal and Rel2 Negatively Regulates Antimicrobial Peptide Expression in the Tobacco Hornworm *Manduca sexta*. *Sci. Rep.* **6**, 20654; doi: 10.1038/srep20654 (2016).

## Supplementary Material

Supplementary Information
