# Supplementary material for: Co-expression of Dorsal and Rel2 Negatively Regulates Antimicrobial Peptide Expression in the Tobacco Hornworm Manduca sexta
Source: Sci Rep. 2016 Feb 5;6:20654. doi: 10.1038/srep20654 (PMC4742911; doi:10.1038/srep20654)
Supplement: Supplementary Information [file srep20654-s1.doc]

**Supplementary Information**

Co-expression of Dorsal and Rel2 Negatively Regulates Antimicrobial Peptide Expression in the Tobacco Hornworm *Manduca sexta*

Xue Zhong^1,+^, Xiang-Jun Rao^2,+^, Hui-Yu Yi^1^, Xin-Yu Lin^1,3^,

Xiao-Hong Huang^1,3^, Xiao-Qiang Yu^1,*^

^1^Division of Molecular Biology and Biochemistry, School of Biological Sciences, University of Missouri-Kansas City, 5007 Rockhill Road, Kansas City, MO 64110, USA.

^2^Department of Entomology, School of Plant Protection, Anhui Agricultural University, Hefei, Anhui 230036, China

^3^College of Animal Science, Fujian Agriculture and Forestry University, Fuzhou, Fujian350002, China

*Correspondence should be addressed to X.Y. (email: [Yux@umkc.edu](mailto:Yux@umkc.edu))

^+^these authors contributed equally to this work

**This file includes:**

Figure S1 and Table S1

**
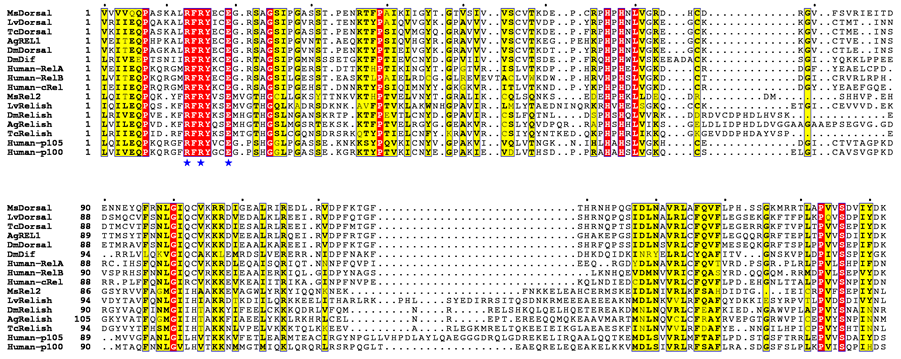

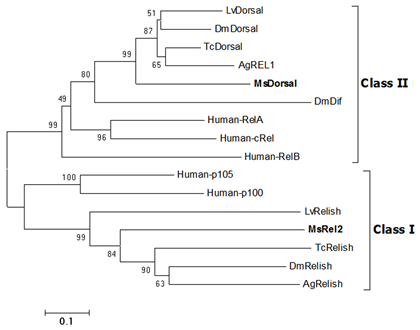

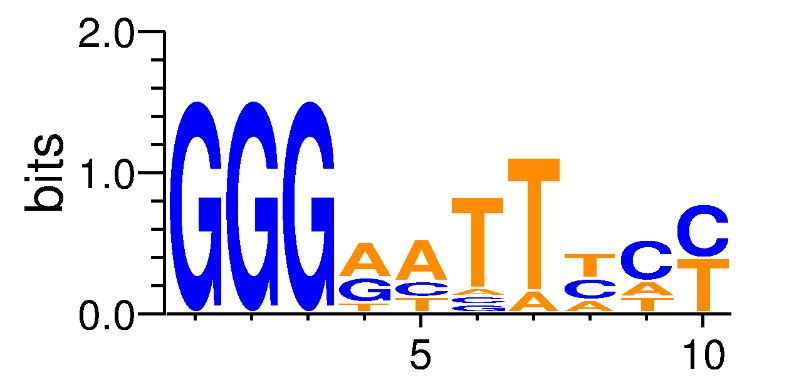
**

**A**

**B**

**C**

**Figure S1. Multiple sequence alignment and phylogenetic tree of the NF-κB Rel-homology domains (RHDs).** The RHD sequences of NF-κB factors were aligned and the alignment was used to generate a neighbor-joining tree (A). Sequence alignment was performed with Clustal Omega (B). Identical residues were shaded in red. Similar residues were shaded in yellow. The residues involved in hydrogen bonds were marked with blue stars below the alignment. Fourteen active NF-κB sites from *M. sexta* and *D. melanogaster* AMP genes were aligned to create a graphical representation with WebLogo3 (C). Ms: *Manduca sexta*; Lv: *Litopenaeus vannamei*; Dm: *Drosophila melanogaster*; Tc: *Tribolium castaneum*; Ag: *Anopheles gambiae* str. PEST. LvDorsal, ACZ98167.1; DmDorsal, AAF53611.1; TcDorsal, EFA02850.1; AgREL1, XP_310177.3; MsDorsal, ADK39025.1; DmDif, AAA28465.1; RelA, NP_068810.3; c-Rel, NP_002899.1; RelB, NP_006500.2; p105, NP_003989.2; p100, NP_001070962.1; LvRelish, ABR14713.1; MsRel2A and MsRel2B, ADK39023.1, ADK39024.1; TcRelish, EEZ97717.1; DmRelish, NP_477094.1; AgRelish, XP_308995.3.

**Table S1. PCR primers used in this study**

| **Primers** | **Forward Primer (5’ 🡪 3’)** | **Primers** | **Reverse Primer (5’ 🡪 3’)** |
| --- | --- | --- | --- |
| **Cloning** | | | |
| Rel2-F1 | **ATTTAAATATAGTTAGACTGAAGTTTAGCGCTCA** | Rel2-R1 | **CGTGAGCGCTAAACTTCAGTCTAACTATATTTA** |
| Dorsal-F1 | **CAATCGCAGCCAATTACGCACAAGGTAAC** | Dorsal-R1 | **GCTTCGCGTAAAATATCTTTTAGTTGTCGATTC** |
| **Real-time PCR** | | | |
| Rel2-qPCR-F1 | **CCGCATAAGCCGTTGTTACG** | Rel2-qPCR-R1 | **TGGTGATTGACGTACGGTGG** |
| Rel2B-qPCR-F2 | **TGACAATGTCCCAGGTTCGG** | Rel2B-qPCR-R2 | **TTCAGCAAAAACAACGGGCG** |
| Dorsal-qPCR-F1 | **TTCGATATGAGTGCGAGGGC** | Dorsal-qPCR-R1 | **CGACACTACGATGGAGACGG** |
| RPS3-F | **GTTGCGAGGTGGTGGTTTC** | RPS3-R | **CCGTTCTTGCCCTGTTGGTC** |
| **Protein expression** | | | |
| Rel2A/2B-pAC5.1-KpnI-N | **CGGGGTACCGGAATTTGCCAATTGGGTGCTACCAATTTAATAAACTCCACTGGCGTTAGTTTCGGCGTTGCTAATGTCACCAGTTTTGGCAACCAAaATGGCCtcctcttgtccaagcgac** | | |
| Rel2A-pAC5.1-ApaI-C | **GAAGGGCCCTAGATTCGTCATGGGTTG** | Rel2B-pAC5.1-ApaI-C | **GAAGGGCCCAAGAATTCTTTTGACATAGTAT** |
| Dorsal-pAC5.1-KpnI-N | **CGGGGTACCCAAaATGGCCCCGTACGTGGTCGTCGTGCAGCAG** | Dorsal-pAC5.1-ApaI-C | **GAAGGGCCCCTTCTTGTCGTAGATGACGTCGGA** |
| Dorsal-pIZ-KpnI-N | **CTTGGTACCAATAAAGTGCGATGGcgCCGTACGTGGTCGTCGTG** | Dorsal-pIZ-XbaI-C | **CCCTCTAGAGTCTTCTTGTCGTAGATGAC** |
| Rel-pIZ-KpnI-N | **CTTGGTACCAATTGCCCTTACCATGGAGAAAAAAATCCCATATCTACAGATCAC** | Rel-pIZ-XbaI-Flag-C | **CCCTCTAGATCACTATTACTTGTCATCGTCGTCCTTGTAGTCCTTCAAATTATAAATGGG** |
| Rel-RHD-EcoRI-N | **CCGGAATTCATGCCATATCTACAGATCACAGA** | Rel-RHD-NotI-Flag-C | **GCGGTTTAAACTCACTTGTCATCGTCGTCCTTGTAGTCAGCGGCCGCCTTCAAATTATAAATGGGTTC** |
| Dorsal-RHD-NcoI-N | **ACGCCATGGCGCCGTACGTGGTCGTCGTG** | Dorsal-RHD-XhoI-C | **GATAAGCTTTTACTTCTTGTCGTAGATGATGAC** |
| **Mutant reporter plasmids** | | | |
| Lysozyme^Moricin-κB5-rev^-N | **CTAAACTAATATTGGGACTTTACTGCCAGTATCAACCTGGATT** | Lysozyme^Moricin-κB5-rev^-C | **ATACTGGCAGTAAAGTCCCAATATTAGTTTAGGTACCTATCG** |
| Lysozyme^Moricin-κB5-GATA-rev^-N | **CTAAACTAATATTCAGATAACGATAGGGACTTTACTGCCAGTATC** | Lysozyme^Moricin-κB5-GATA-rev^-C | **GTAAAGTCCCTATCGTTATCTGAATATTAGTTTAGGTACCTATCG** |
| Lysozyme^Moricin-κB5^ -N | **CTAAACTAATATTGTAAAGTCCCTGCCAGTATCAACCTGGATT** | Lysozyme^Moricin-κB5^-C | **ATACTGGCAGGGACTTTACAATATTAGTTTAGGTACCTATCG** |
| Lysozyme^Moricin-κB5-GATA^-N | **ATTGTAAAGTCCCTATCGTTATCTGTGCCAGTATCAACCTGGAAT** | Lysozyme^Moricin-κB5-GATA^-C | **ATACTGGCACAGATAACGATAGGGACTTTACAATATTAGTTTAG** |
| Moricin^Lysozyme-κB-rev^-N | **GCCTTTGTAAATTAGAAAGTCCCTATCGTTATCTGAGAGTATAAA** | Moricin^Lysozyme-κB-rev^-C | **GATAACGATAGGGACTTTCTAATTTACAAAGGCCCGGAATTAC** |
| Moricin^Lysozyme-κB^ -N | **GCCTTTGTAAATTGGGACTTTCTTATCGTTATCTGAGAGTATAAA** | Moricin^Lysozyme-κB^ -C | **GATAACGATAAGAAAGTCCCAATTTACAAAGGCCCGGAATTAC** |
| Moricin^Lysozyme-κB-rev-ΔGATA^-N | **GCCTTTGTAAATTAGAAAGTCCCTATAATTATATTAGAGTATAAA** | Moricin^Lysozyme-κB-rev-ΔGATA^-C | **TATAATTATAGGGACTTTCTAATTTACAAAGGCCCGGAATTAC** |
| Moricin^Lysozyme-κB-ΔGATA^-N | **GCCTTTGTAAATTGGGACTTTCTTATAATTATATTAGAGTATAAA** | Moricin^Lysozyme-κB-ΔGATA^-C | **TATAATTATAAGAAAGTCCCAATTTACAAAGGCCCGGAATTAC** |
